# Identification of the phytobioactive *Polygonum cuspidatum* as an antiviral source for restricting dengue virus entry

**DOI:** 10.1038/s41598-020-71849-3

**Published:** 2020-10-02

**Authors:** Yu-Ting Kuo, Ching-Hsuan Liu, Jin-Wei Li, Chien-Ju Lin, Alagie Jassey, Huey-Nan Wu, Guey Chuen Perng, Ming-Hong Yen, Liang-Tzung Lin

**Affiliations:** 1 Department of Medical Imaging, Chi Mei Medical Center, Tainan, Taiwan; 2 Graduate Institute of Medical Sciences, College of Medicine, Taipei Medical University, Taipei, Taiwan; 3 Department of Microbiology & Immunology, Dalhousie University, Halifax, NS Canada; 4 Graduate Institute of Natural Products, College of Pharmacy, Kaohsiung Medical University, Kaohsiung, Taiwan; 5 School of Pharmacy, College of Pharmacy, Kaohsiung Medical University, Kaohsiung, Taiwan; 6 International Ph.D. Program in Medicine, College of Medicine, Taipei Medical University, Taipei, Taiwan; 7 Institute of Molecular Biology, Academia Sinica, Taipei, Taiwan; 8 Department of Microbiology and Immunology & Institute of Basic Medical Sciences, College of Medicine, National Cheng Kung University, Tainan, Taiwan; 9 Center of Infectious Diseases and Signaling Research, National Cheng Kung University, Tainan, Taiwan; 10 Department of Microbiology and Immunology, School of Medicine, College of Medicine, Taipei Medical University, Taipei, Taiwan

**Keywords:** Dengue virus, Antiviral agents

## Abstract

Dengue virus (DENV) is a mosquito-borne pathogen that is becoming a serious global threat, owing to its rising incidence in inter-tropical regions that yield over 50 million annual infections. There are currently no approved antiviral agents for the management of dengue, and recent shortcomings in its immunization called for immediate action to develop effective drugs with prophylactic ability to better manage its infection. In an attempt to discover novel antiviral sources, we identified the medicinal herb *Polygonum cuspidatum* (PC) as a bioactive botanical material against DENV infectivity. Specifically, the methanolic extract from PC rhizomes (PCME) potently inhibited DENV infection without causing significant cytotoxicity. Further examination on the viral life cycle demonstrated that PCME particularly targeted the initial stages of DENV infection, while pre- and post-infection treatments had no effect. More importantly, the PCME could efficiently inactivate DENV free virus particles and block the viral attachment and entry/fusion events without apparently influencing viral replication, egress, and cell-to-cell spread. The antiviral effect of PCME was also recapitulated in infection analysis using DENV pseudoparticles displaying viral structural proteins that mediate DENV particle entry. Besides, PCME treatment also inhibited direct DENV entry into several cell types relevant to its infection and reduced viral infectivity of other members of the *Flaviviridae* family, including the hepatitis C virus (HCV) and Zika virus (ZIKV). Due to its potency against DENV entry, we suggest that the phytobioactive extract from PC is an excellent starting point as an antiviral source material for further development of therapeutic strategies in the prophylactic management of DENV infection.

## Introduction

Dengue virus (DENV) is an enveloped, positive sense single-strand RNA virus belonging to the *Flaviviridae* family^1^, which includes important human viruses such as hepatitis C virus (HCV), Zika virus (ZIKV), and West Nile virus (WNV). The virus is primarily transmitted by *Aedes aegypti* mosquito bites and is serologically divided into four serotypes (DENV-1 through 4). Translation of its 11 kb genome yields 3 structural proteins for forming the basic viral particle including capsid (C), precursor membrane (prM), and envelope (E) glycoprotein, and 7 nonstructural (NS) proteins including NS1, NS2A, NS2B, NS3, NS4A, NS4B, and NS5, which mainly participate in viral replication^2^.

DENV infection symptoms can range from mild undifferentiated febrile syndromes (dengue fever) to life-threatening dengue hemorrhagic fever or dengue shock syndrome. The World Health Organization (WHO) has recently observed a sharp increase in DENV infection (50–100 million new cases annually) with 500,000 people per year suffering from severe dengue at a 2.5% deaths rate, which causes serious economic and public health burden^3,4^. Secondary infection by a different DENV serotype could lead to severe clinical manifestations due to antibody-dependent enhancement (ADE) of DENV infection by pre-existing non-neutralizing antibodies^5^, posing one of the current challenges to developing a safe and effective dengue vaccine^1^. Although a recently licensed vaccine (Dengvaxia®; Sanofi Pasteur) could provide protection in individuals previously infected with DENV^6^, it posed safety concerns in increasing deadly complications in individuals vaccinated without prior DENV infection^7^. At present, no other licensed antiviral drugs are available to combat dengue diseases, and supportive care such as analgesics, fluid replacement, and bed rest with continuous monitoring of symptoms are the main treatments for DENV infection. Thus, identification of effective starting-point antivirals agents/source materials and treatment strategies is urgently needed to address the expanding dengue epidemic.

Plants and their phytochemical contents are an important source of novel antiviral drug discovery owing to its biodiversity. *Polygonum cuspidatum* Sieb. et Zucc. (*Polygonum cuspidatum*, PC), belongs to the Polygonaceae (‘buckwheat’) plant family, and is a popular oriental medicinal herb that has been documented to possess anti-inflammatory and antiviral properties^8,9^. Specifically, crude PC extract and its major components have been observed to exert inhibitory activities against several viruses such as herpes simplex type 1^10^, Epstein-Barr virus^11^, Coxsakievirus B4^12^, influenza virus^13^, hepatitis B virus^14^, and human immunodeficiency virus 1^15^. However, the antiviral activity of PC on DENV infection is unknown. In order to expand the scope of antiviral sources for developing anti-DENV therapies, we investigated in this study the impact of the methanolic extract of PC (PCME) on DENV life cycle by mechanistically dissecting the steps of viral infection. We also examined its activity against different cell types relevant to DENV infection and its effect against other members of the *Flaviviridae* family. Herein, we describe our identification of PCME as a potent bioactive botanical source material against DENV infection.

## Materials and methods

### Drug material preparation

Air-dried rhizomes from *Polygonum cuspidatum* Sieb. & Zucc. (PC; LSID#695612-1 from the International Plant Names Index [IPNI]^16^) were obtained from local pharmacy store (Kaohsiung, Taiwan) and authenticated by Dr. Ming-Hong Yen anatomically as well as by high performance liquid chromatography analysis through comparison to known molecular standards as previously described^17^. A voucher specimen (CTM-PPC02) was prepared and deposited at the Kaohsiung Medical University Herbarium. The plant material was extracted using methanol as previously described^14^ to obtain the methanol-isolated PC (PCME). The lyophilized sample was dissolved in dimethyl sulfoxide (DMSO; Sigma, St. Louis, MO, USA) for treatment analysis. The final concentration of DMSO in the samples during treatment in all experiments was < 1%.

### Cell culture and virus production

African green monkey kidney Vero (ATCC CCL-81) and the human hepatoma Huh-7 cells were cultured in Dulbecco’s Modified Eagle’s Medium (DMEM; GIBCO-Invitrogen, Carlsbad, CA, USA). The media were supplemented with 10% fetal bovine serum (FBS; GIBCO-Invitrogen) and 1% Penicillin–Streptomycin-Amphotericin B Solution (Biological Industries; Cromwell, CT, USA). The Huh7.D2-FLuc-SGR-Neo cells, which are Huh-7 hepatoma cells established with replicating DENV-2 subgenomic RNA, D2-FLuc-SGR-Neo^18^, were maintained in the above described media plus 1 mg/ml G418 (Sigma). DENV-2 (strain 16681) stocks were produced and titered as previously reported in Vero cells by immunohistochemical staining plaque assay^19^. Virus concentration is expressed as plaque forming units (PFU) per well or multiplicity of infection (MOI). The C6/36 *Aedes albopictus* mosquito cells were cultured in Minimum Essential Medium alpha (α-MEM; GIBCO-Invitrogen) containing 10% FBS with incubation at 28 °C, and the K562 (erythro-megakaryoblastic leukemia) and U937 (monocytic leukemia) suspension cells were cultured in RPMI-1640 (GIBCO-Invitrogen) at 37 °C in a humidified incubator containing 5% CO_2_. The cell culture-derived infectious HCV particles (HCVcc) expressing a *Gaussia* luciferase reporter were produced by electroporating Huh-7 cells with the genotype 2a Jc1FLAG2 (p7-nsGluc2A) construct as previously described^20^. The WNV reporter virus particles (WNV.RVPs) expressing a *Renilla* luciferase were generated as reported previously^21^. The DENV pseudoparticles (DENVpp) consisting of DENV structural proteins (strain 16681) that package a *Renilla* luciferase-tagged WMV backbone were produced using a previously described approach^22^. The recombinant Zika virus infectious clone particles (ZIKVic) were produced using previously described methods^23,24^. The respective medium containing 2% FBS for the above-mentioned cell types was used as the basal medium for all viral infection analyses.

### Cytotoxicity analysis

Vero cells were treated with different drug concentrations (10, 25, 50, 100, and 250 μg/ml) for 6 days followed by cell viability analysis using XTT (2,3-bis[2-methoxy-4-nitro-5-sulfophenyl]-5-phenylamino)-carbonyl]-2H-tetrazolium hydroxide) (Sigma) as previously described^25^. The concentration of 50% cellular cytotoxicity (CC_50_) of the test samples was calculated using GraphPad Prism software (San Diego, CA, USA).

### Dose–response antiviral assay

Antiviral effect against DENV infection was evaluated by immunohistochemical staining plaque assay^19^. Briefly, Vero cells (2 × 10^5^ cells/well of 12-well plates) were inoculated with DENV-2 (100 PFU/well) in the presence of various concentrations of the test drugs (1, 5, 10, 20, and 30 μg/ml) for 1.5 h at 37 °C. Cells were subsequently washed with PBS twice and overlaid with 2% FBS DMEM containing 0.75% methylcellulose and the respective drug concentrations for 6 days. At the end of the incubation, the overlay media were removed, and the cells were PBS-washed, fixed with ice-cold methanol, and blocked with 3% bovine serum albumin (BSA) in PBS. Viral plaques were immunohistochemically stained using anti-Flavivirus Group Antigen antibody (1:1,000; Millipore, Billerica, MA, USA) and goat anti-mouse IgG (H + L) alkaline phosphatase (AP)-conjugated antibody (1:5,000; Invitrogen), followed by development with Vector Black AP Substrate Kit (Vector Laboratories; Burlingame, C, USA). Viral infectivity (%) was calculated relative to control (DMSO) treatment and the 50% effective concentration (EC_50_) of the drug samples’ antiviral activity was determined as reported^25^.

### Time-of-drug-addition assay

The time-of-drug-addition assay was carried out as previously reported^25^ to specifically examine the influence of drug pretreatment of the host cells, adding the drugs and virus simultaneously (coaddition), and postinfection treatment (Fig. 2A). For all analyses, DENV-2 (inoculated at 100 PFU/well) viral plaques were examined in Vero cells (2 × 10^5^ cells/well of 12-well plates) by immunohistochemical staining as described above.

### Synchronized infection analysis on early viral entry

Analysis of drug effect in inactivating cell-free virus particles, perturbing viral attachment, and blocking entry/fusion were conducted as previously described^19^ (Fig. 3A). Viral infectivity (%) was determined by analyzing the resulting DENV-2 (inoculated at 100 PFU/well) plaques in Vero cells (2 × 10^5^ cells/well of 12-well plates) by immunohistochemical staining as described above.

### Flow cytometry-based viral infection assay

Vero (1 × 10^6^ cells/well), Huh-7 (5 × 10^5^ cells/well), C6/36 (1 × 10^6^ cells/well), K562 (1 × 10^6^ cells/well), or U937 (1 × 10^6^ cells/well) in 6-well plates were infected with DENV-2 (Vero and C6/36: MOI = 0.1; Huh-7, K562, and U937: MOI = 1) in the presence of the drug treatment for 1.5 h. For the monocytic U937 and erythroid K562 cells that express Fcγ receptors on their cell surface and whose low permissiveness to DENV infection could be increased via antibody-dependent enhancement^26,27^, subneutralizing concentration (1: 100) of the anti-Flavivirus Group Antigen 4G2 antibody (Millipore) was additionally added to the viral inoculum (with or without PCME treatment) during the infection as previously described for both cell types^26,28,29^. The cells were then washed and incubated for 3 days before being harvested with cell dissociation buffer (Sigma) and fixed with 4% formaldehyde. Following fixation, cells were PBS washed, permeabilized using 0.1% Triton X-100, and blocked with 3% BSA in PBS before staining with anti-Flavivirus Group Antigen 4G2 antibody (1:2000; Millipore) and Alexa Fluor 488 goat anti-mouse IgG (H + L) (1:1,000; Invitrogen). Flow cytometry analysis was carried out using a Cytomics FC500 Flow Cytometer (Beckman Coulter; Indianapolis, IN, USA) and the CXP analysis software (Beckman Coulter).

### qPCR analysis of DENV RNA

The qPCR analysis of intracellular viral RNA was performed following a previously described method^30^ with some modifications. Briefly, Vero cells (1 × 10^6^ cells/well of 6-well plates) were infected with DENV-2 (MOI = 0.1) at 37 °C for 1.5 h, and then washed with PBS before drug treatment in media for 3 days. Total cellular RNA was extracted using the TRIzol reagent (Invitrogen) according to the manufacturer’s instructions. For qPCR, RevertAid First Strand cDNA Synthesis Kit (Thermo Fisher Scientific; Waltham, MA, USA) was used for first-strand synthesis, and a 20 μl reaction solution containing 50 ng cDNA and Maxima SYBR Green qPCR master mix (Thermo Fisher Scientific) was prepared for cDNA amplification using primers targeting the DENV-2 NS5^31^ (forward primer: GGAAGGAGAAGGACTGCACA; reverse primer: ATTCTTGTGTCCCATCCTGCT). Samples were analyzed on an Applied Biosystems® 7,500 System (Applied Biosystems; Foster City, CA, USA) and results were determined using the associated software.

### Subgenomic DENV replicon test

The Huh7.D2-FLuc-SGR-Neo cells, which harbor DENV-2 subgenomic RNA D2-FLuc-SGR-Neo tagged with firefly luciferase reporter and the neomycin phosphotransferase gene^18^, were seeded (1 × 10^4^ cells/well of 96-well plates) to obtain monolayers and then treated with the drug samples for 3 days. Cell lysates were collected at the end of the incubation and then assessed for firefly luciferase activity using the Luciferase Assay System (Promega; Madison, WI, USA) and a luminometer (Promega). DENV-2 subgenome replication was expressed as log_10_ of relative light units (RLU). Ribavirin (Sigma) was included as positive control.

### Western blot analysis

Vero cells (1 × 10^6^ cells/well of 6-well plates) were infected with DENV-2 (MOI = 0.1) at 37 °C for 1.5 h, and then washed with PBS before treatment with the test drug in media for 3 days. For total protein lysates, supernatants from virus-infected cells were removed and the cells were washed and harvested using RIPA Buffer (Millipore; Billerica, MA, USA) containing protease inhibitors (Roche Molecular Biochemicals; Indianapolis, IN, USA). To isolate DENV particles released into the media from DENV-infected cells, the collected supernatants were centrifuged to remove cell debris. The clarified supernatants were then treated with polyethylene glycol (PEG)-8,000 (8% w/v) at 4 °C overnight before further centrifugation to obtain the virus pellet for protein extraction using RIPA buffer^32^. Protein samples were analyzed by standard Western immunoblotting using anti-Flavivirus Group Antigen 4G2 (targeting E glycoprotein, 1:3,000; Millipore), anti-Dengue virus NS1 (1: 500; Genetex, Irvine, CA, USA), anti-Dengue virus Type 2 NS5 (1: 1,000; Genetex), and β-actin (1:10,000; Cell Signaling Technologies, Danvers, MA, USA) antibodies. Protein detection was performed using horseradish peroxidase (HRP)-conjugated anti-rabbit (for NS1) or anti-mouse (for NS5 and E) secondary antibodies (Thermo Fisher Scientific) and Immobilon™ Western Chemiluminescent HRP Substrate solution (Millipore). Protein bands were visualized on a UVP imager (UVP; Upland, CA, USA) and densitometry was analyzed by ImageJ Software (developed by W. Rasband; National Institutes of Health, Bethesda, MD, USA).

### Viral cell-to-cell spread analysis

Vero cells (1 × 10^6^ cells/well) were seeded on cover glass (Deckgläser; Thermo Fisher Scientific) in 6-well plates. Cells were infected with DENV-2 (MOI = 0.1) at 37 °C for 1.5 h, and then washed with PBS before drug treatment for 3 days (for the 72 h virus only group) or 6 days (for all other groups). Cells were then PBS-washed, fixed with 4% formaldehyde, permeabilized using 0.1% Triton X-100, and blocked with 3% BSA in PBS. The DENV-positive foci were analyzed by immunofluorescence staining of the cover glass using primary anti-Flavivirus Group Antigen 4G2 antibody (1:1,000; Millipore) and Alexa Fluor 488 anti-mouse IgG (H + L) secondary antibody (1:500; Invitrogen), followed by treatment with DAPI Fluoromount-G (SouthernBiotech; Birmingham, AL, USA) and sealing on the Frosted Microscope Slides (Thermo Fisher Scientific) with nail polish. Visualization was carried out with a ZEISS LSM 700 confocal laser scanning microscope (ZEISS International; Jena, Germany). Three random viral foci in each treatment group were assessed to measure their diameter as well as determine the number of cells per DENV E-positive focus.

### Analysis of PCME’s effect against infectivity of DENVpp, HCV, ZIKV, and WNV

For assessing the antiviral activity of PCME against DENVpp, HCV, ZIKV, and WNV, Vero cells (ZIKV: 1 × 10^6^ cells/well of 6-well plates; DENVpp and WNV: 1 × 10^4^ cells/well of 96-well plates) and Huh-7 cells (HCV: 1 × 10^4^ cells/well of 96-well plates) were seeded overnight and infected with the respective viral inoculums (DENVpp, HCVcc, ZIKVic, and WNV.RVP) in the presence or absence of PCME at non-cytotoxic concentrations. The cells were then washed with PBS and incubated with DMEM containing 2% FBS for 72 h. At the end of the incubation, cells were washed, and wells in the HCV infectivity experiment were analyzed for luciferase activity using the Pierce *Gaussia* Luciferase Flash Assay Kit (Pierce; Rockford, IL, USA), while wells from the DENVpp and WNV infectivity experiments were assayed using the Promega *Renilla* Luciferase Assay System (Promega), before obtaining readouts on a luminometer (Promega). For the ZIKV infectivity experiment, cells were collected by trypsinization and processed as described in the flow cytometry-based viral infection assay section using the anti-Flavivirus Group Antigen 4G2 antibody (1:2000; Millipore) followed by Alexa Fluor 488 goat anti-mouse IgG (H + L) (1:1,000; Invitrogen) as probes.

### Statistical analysis

Statistical analysis was performed by one-way ANOVA with Dunnett’s multiple comparisons test. Comparisons were made with the DMSO control treatment group unless otherwise specified, and a *P* value < 0.05 was considered as statistically significant. All data were presented as means ± standard errors of the means (SEM) from three independent experiments (n = 3).

## Results

### PCME dose-dependently inhibits DENV-2 infection

**Figure 1:**
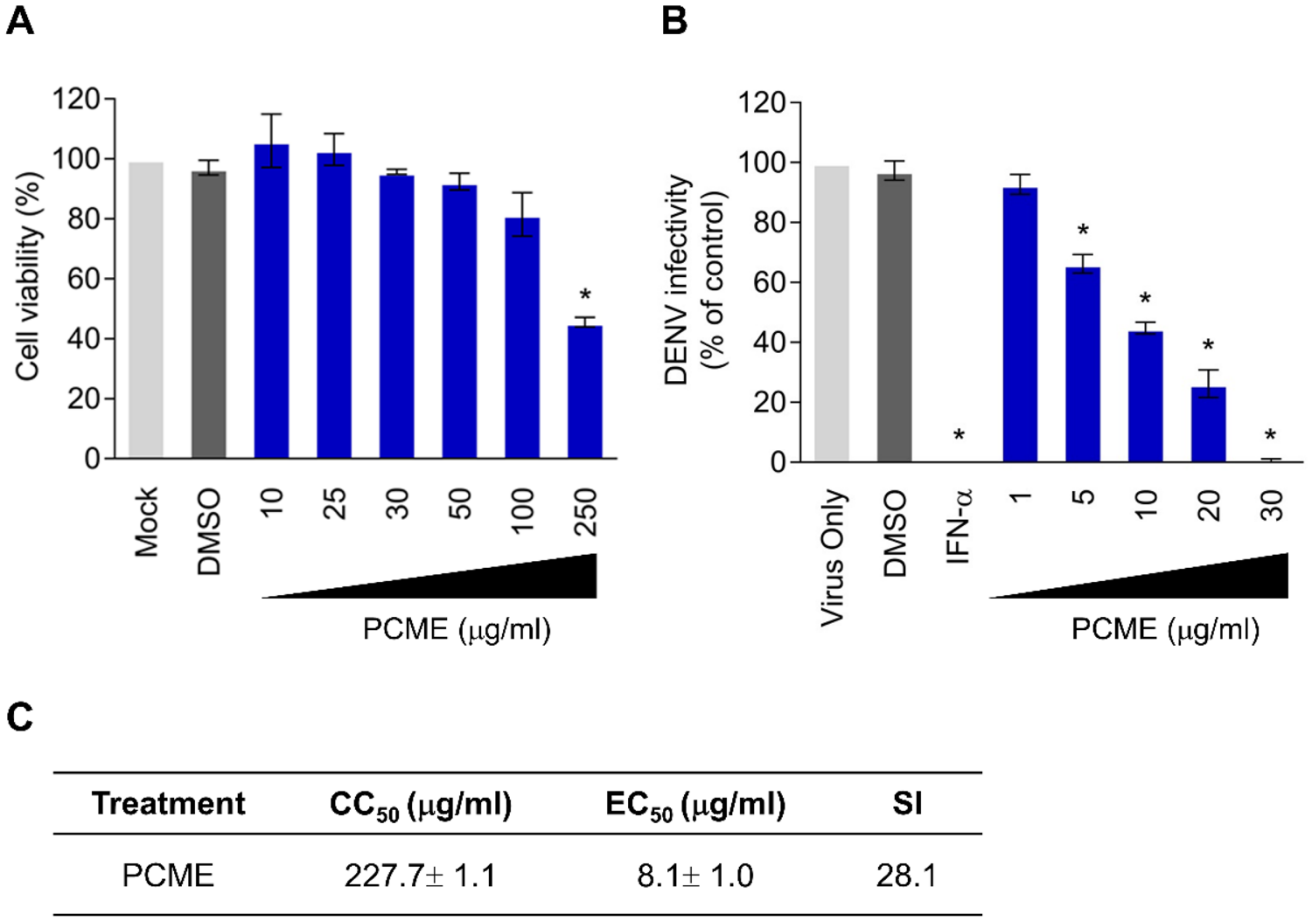
Antiviral activity of PCME against DENV infection. **(A)** Cytotoxicity of PCME on Vero cells as analyzed by XTT cell viability assay. DMSO (1%) served as negative control treatment. **(B)** Immunostained plaque assay-based antiviral dose response analysis of PCME treatments against DENV infection (100 PFU/well). DMSO (0.06%) served as negative control and data is expressed as percent (%) DENV infectivity. **(C)** The 50% cytotoxic concentration (CC_50_), 50% effective concentration (EC_50_), and selectivity index (SI = CC_50_/EC_50_) values of PCME against DENV infection. Data shown are means ± SEM (*P < 0.05) from 3 independent experiments.

In order to identify novel antiviral sources for the development of anti-DENV therapies, we evaluated the influence of the methanolic extract (PCME) from PC, a plant known to possess antiviral potency, against DENV infection. Vero cells, a gold-standard tool in DENV antiviral investigations, were infected with serotype 2 DENV in the presence or absence of test drugs at various doses. A cell viability analysis on naïve Vero cells was concomitantly carried out at the same dose range. PCME exhibited a dose-dependent inhibition on the DENV infection, with a concentration up to 30 μg/ml completely abrogating the viral infection without inducing significant cytotoxicity (Fig. 1A,B). The 50% cytotoxicity (CC_50_), effective antiviral concentration (EC_50_), and selectivity index (SI; CC_50_/EC_50_) values were determined to be CC_50_ = 227.7 ± 1.1; EC_50_ = 8.1 ± 1.0; SI = 28.1 (Fig. 1C). The data demonstrated that PCME could efficiently inhibit DENV infection. A concentration of 30 μg/ml was chosen for all subsequent experiments.

### Antiviral activity of PCME occurs specifically during DENV particle infection

**Figure 2:**
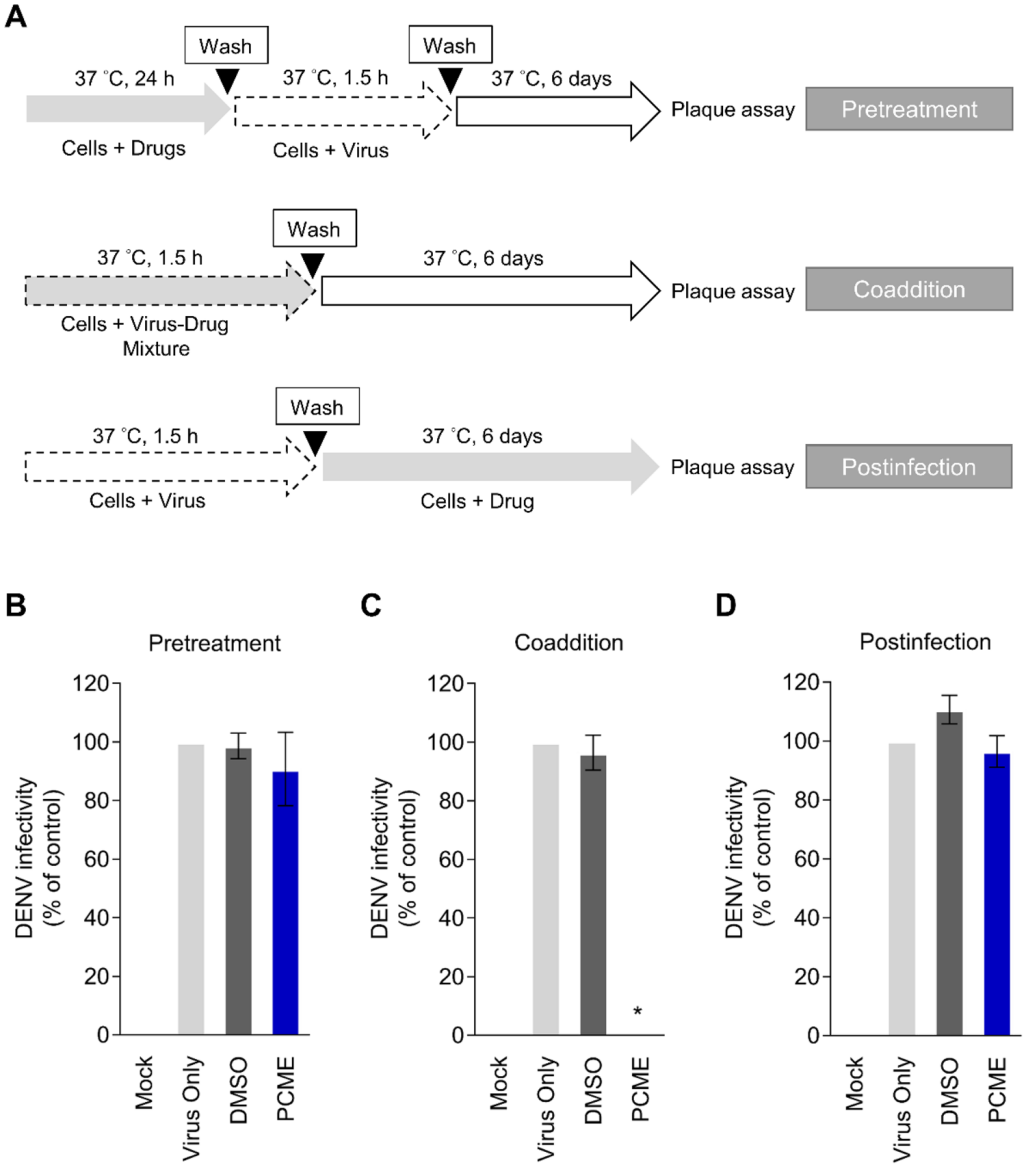
PCME targets the early phase of DENV infection. **(A)** Schematics of the time-of-drug-addition analysis. **(B)** Effect of PCME pretreatment on cells prior to DENV infection. **(C)** Coaddition treatment analysis of PCME during DENV infection of cells. **(D)** Postinfection treatment effect of PCME on DENV-infected cells. Results were obtained using immunostained plaque assay in Vero cells following 6 days of incubation. PCME = 30 μg/ml; DENV = 100 PFU/well; DMSO = 0.06%. Data shown are means ± SEM (*P < 0.05) from 3 independent experiments.

To investigate the target window from the antiviral activity of PCME treatment, a time-of-drug-addition assay was performed by adding the drug before, during, or after viral inoculation (Fig. 2A). The results showed that while pretreatment (Fig. 2B) and postinfection (Fig. 2D) treatment with PCME had little influence on the resulting DENV infection, the test drug displayed robust antiviral activity when the virus and PCME were simultaneously present during cotreatment on the host cells (Fig. 2C). Thus, PCME appeared to specifically target DENV infection during the early stages of the viral life cycle.

### PCME inhibits multiple steps of DENV early viral entry

**Figure 3:**
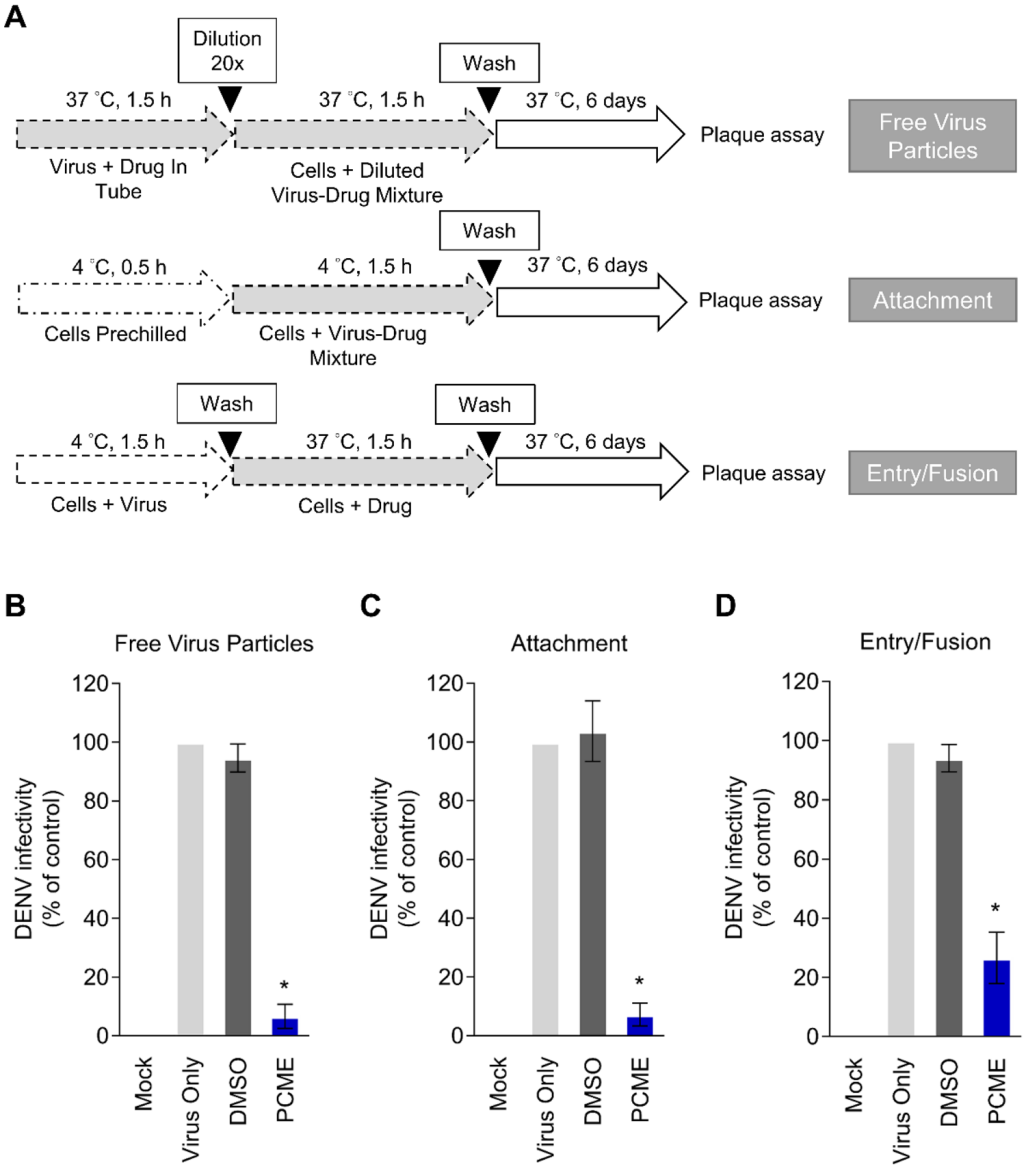
PCME inactivates free DENV particles and blocks viral attachment and entry/fusion steps. **(A)** Schematics of the synchronized infection analysis on DENV early viral entry. **(B)** Treatment effect of PCME on cell-free DENV particles. **(C)** Effect of PCME on DENV attachment. **(D)** Influence of PCME on DENV entry/fusion. All data were obtained by immunostained plaque assay in Vero cells at the end-point (6 days) of the experiments. PCME = 30 μg/ml; final DENV concentration = 100 PFU/well; DMSO = 0.06%. Data shown are means ± SEM (*P < 0.05) from 3 independent experiments.

The initial steps of viral infection are mediated by viral particle interaction with the host cells, which leads to viral entry. To further investigate how PCME inhibits the early stages of DENV infection, we explored its treatment impact on DENV early entry events. We first tested the ability of PCME in inactivating/neutralizing viral particles by incubating DENV virions with the test drug under cell-free condition, followed by dilution of the virus-drug mixture to an ineffective drug concentration (Fig. 3A), which prevents meaningful interaction between the drug and the host cells^33^. As shown in Fig. 3B, PCME effectively diminished the infectivity of cell-free DENV virions, suggesting that it can render the DENV particles non-infectious.

Next, we assessed the impact of PCME on DENV attachment. Cells were first concurrently treated with the DENV particles and the test drug at 4 °C (Fig. 3A), which allows for virus binding but precludes viral internalization^33^, and then subsequently incubated at 37 °C to induce viral entry. While the control DMSO treatment resulted in similar viral plaque formation relative to the DENV only group, PCME treatment significantly abrogated the DENV infectivity (Fig. 3C). The results therefore demonstrated that PCME strongly inhibited DENV binding to the host cell.

Following the above observations, we then sought to examine the influence of PCME on DENV particles’ post-binding viral entry/fusion step. For this purpose, DENV particles were allowed to first attach to the target cell surface at 4 °C before adding the test drug and shifting to 37 °C to facilitate viral entry/fusion (Fig. 3A). As depicted in Fig. 3D a significant drop in DENV infectivity occurred with PCME treatment, revealing that the drug could also strongly inhibit the post-attachment DENV entry/fusion steps, albeit to a lesser magnitude than its effect on the free viral particles and viral attachment.

**Figure 4:**
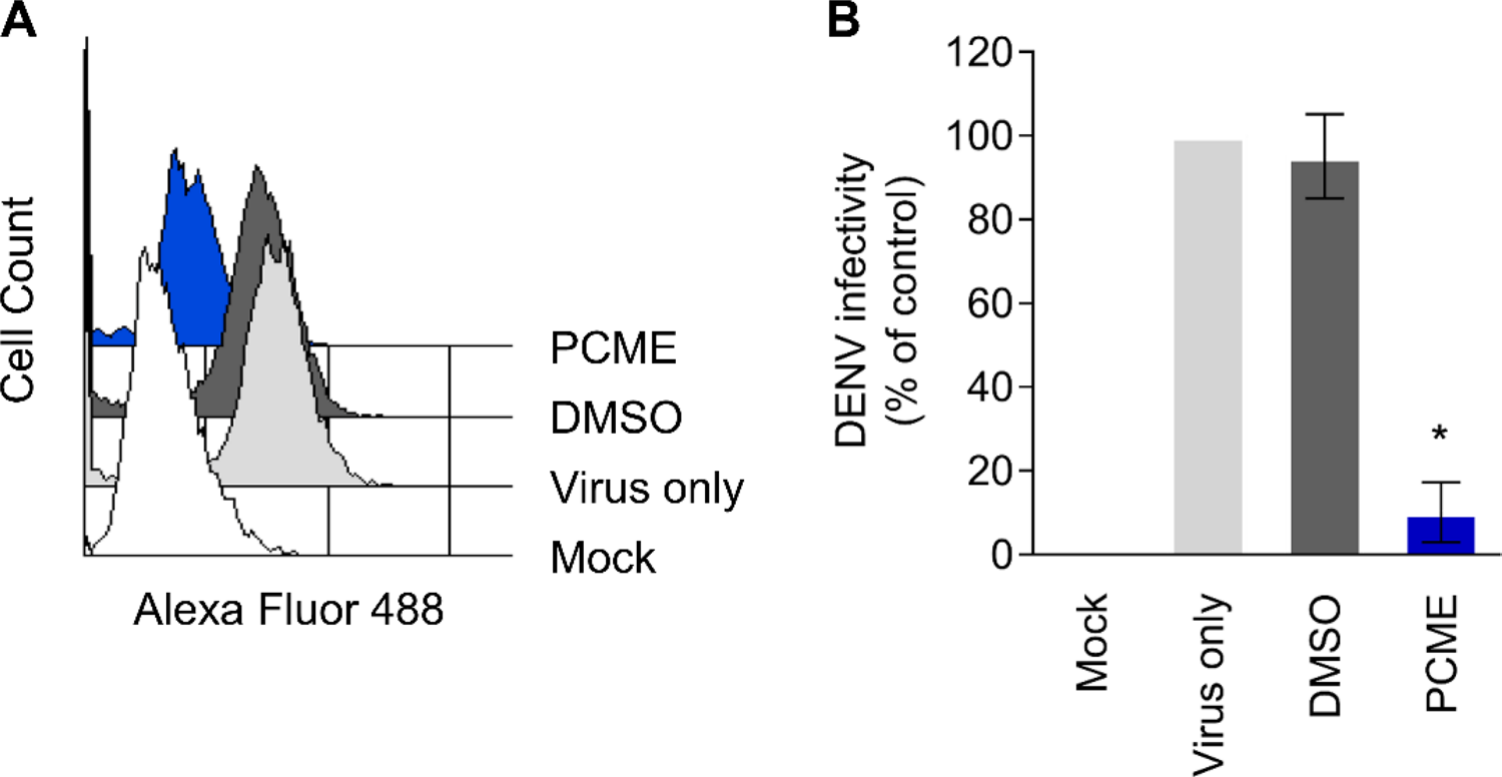
Validation of PCME-mediated inhibition on DENV viral entry using flow cytometry. **(A)** Effect of PCME treatment on DENV viral infection of host cells. Cells were infected with DENV in the presence of the test drug and subjected to flow cytometry detection of intracellular DENV E protein expression. **(B)** Analysis of mean fluorescence intensity from (A). Data shown are means ± SEM (*P < 0.05) or representative histogram from 3 independent experiments.

To further substantiate the influence of PCME on precluding DENV early entry steps, we conducted a flow cytometry-based viral infection assay. Vero cells were inoculated with DENV with concurrent addition of PCME or DMSO control for 1.5 h, before washing with PBS followed by 72 h of incubation prior to detecting intracellular DENV E protein production using flow cytometry. Results in Fig. 4A and its accompanying mean fluorescence analysis (Fig. 4B) demonstrated that, in contrast to the DMSO control, which did not significantly alter the viral protein expression (hence the viral infectivity), PCME treatment robustly precluded DENV infection to the host cells, yielding low levels of DENV protein detected. This supports the finding that PCME exerts its antiviral effects against DENV by blocking the early viral entry stages.

### PCME blocks host cell infection mediated by DENV surface structural proteins displayed on viral pseudoparticles

**Figure 5:**
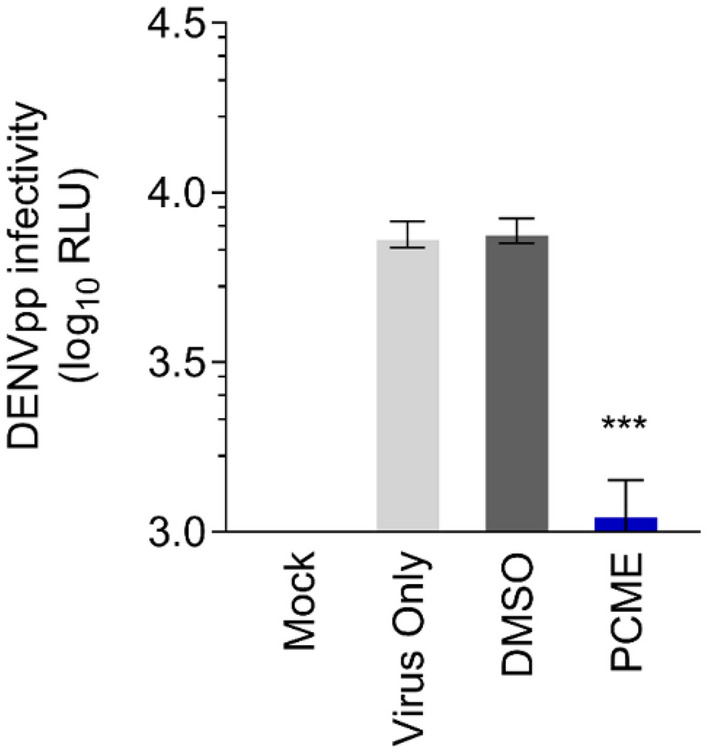
PCME impedes DENVpp entry. Vero cells were concurrently treated with DENVpp (MOI = 0.1) and PCME (30 μg/ml) for 2 h. The cells were subsequently washed and incubated in basal media at 37 °C for 72 h, followed by luciferase reporter activity measurement. Data shown are means ± SEM (*P < 0.05) from 3 independent experiments.

Until now, our results demonstrated that PCME blocks DENV infectivity by targeting the early steps of DENV entry, including inactivating the cell-free viral particles and impeding viral attachment and entry/fusion to the host cells. To validate the antiviral impact of PCME on DENV entry and to determine whether this is mediated by targeting the DENV surface structural proteins, we next assessed the influence of PCME treatment against DENV pseudoparticles (DENVpp). For this purpose, we generated a reporter-tagged DENVpp with a WNV genetic backbone but displaying the DENV M and E on its surface, which form the particle’s outermost architecture and participate in DENV entry^2,34^. This viral pseudotype is capable of a single round infection, and detection of the reporter signal is indicative of successful viral particle entry mediated by the displayed surface structural components^35^. Vero cells were infected with DENVpp in the presence or absence of PCME followed by washing and further incubation before measuring the luciferase reporter activity. Compared to the DMSO control, which had no significant effect on the infectivity of the DENVpp, PCME treatment significantly impeded the DENVpp infectivity in the Vero cells, as indicated by the considerable decrease in the luciferase reporter activity in the presence of the extract (Fig. 5). This result suggests that the antiviral effect of PCME against DENV entry likely involves targeting of the DENV surface structural proteins.

### PCME does not perturb DENV replication, translation, or release

**Figure 6:**
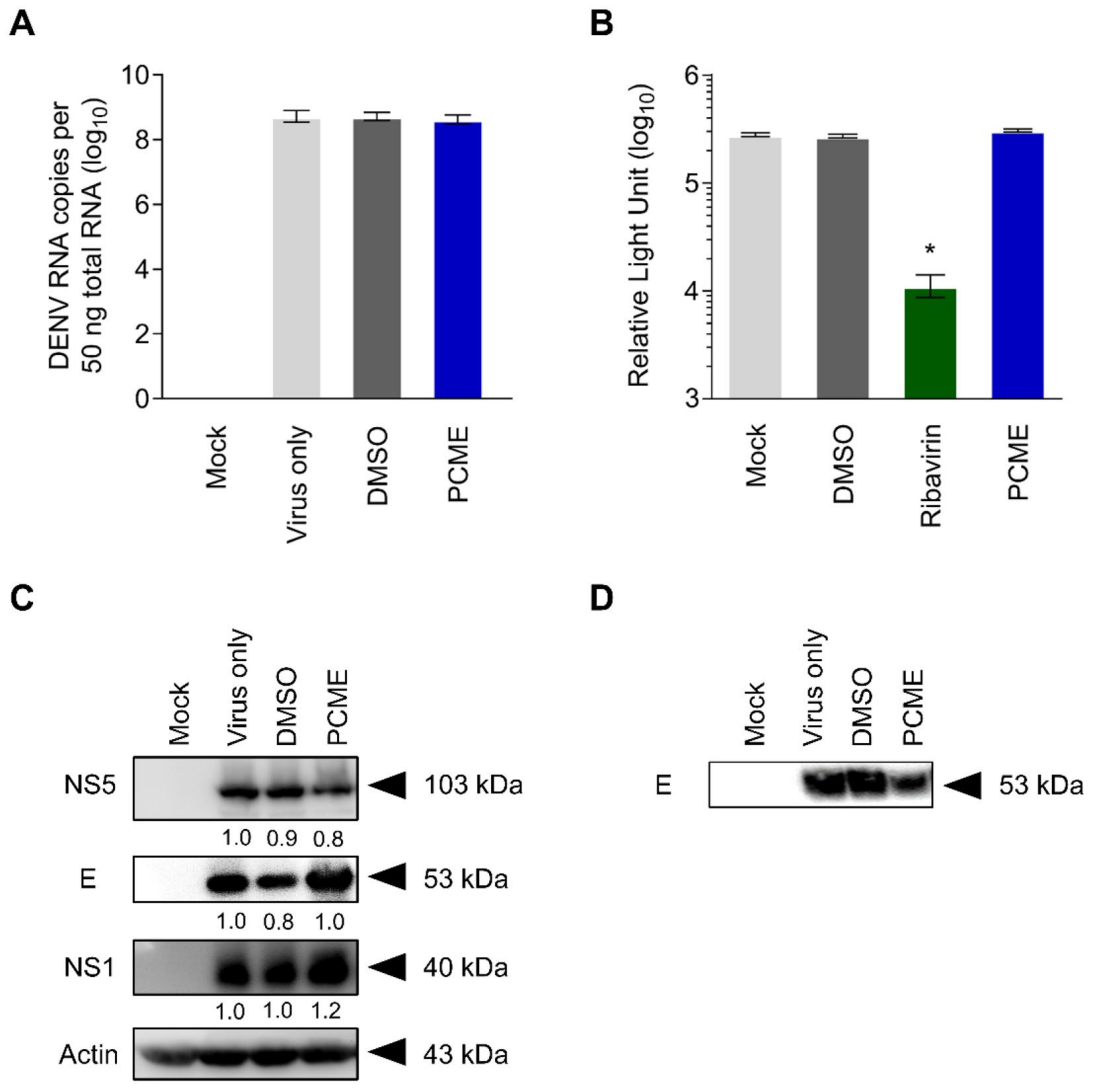
Antiviral activity of PCME treatment is not mediated through influence on DENV replication, translation, or virion release. **(A)** Treatment effect of PCME on DENV intracellular replication. DENV genomic RNA was extracted from DENV-infected Vero cells following 3 days of drug treatment and quantitated using qRT-PCR; DENV MOI = 0.1. **(B)** Luciferase reporter analysis reflecting viral replication/translation from subgenomic DENV replicon Huh7.D2-FLuc-SGR-Neo cells treated with PCME for 3 days. Data are presented in log_10_ of relative light units. **(C)** Effect of PCME on DENV proteins expression from Western blotting analysis of lysates obtained from DENV-infected Vero cells following 3 days of drug treatment. DENV MOI = 0.1; β-actin served as loading control. **(D)** Effect of PCME on DENV release via Western blotting analysis of supernatants from DENV-infected cells after 3 days of drug treatment. PCME = 30 μg/ml; Ribavirin = 200 μM; DMSO = 0.06%. Data shown are means ± SEM (*P < 0.05) or representative immunoblots from 3 independent experiments.

To rule out additional antiviral activities from PCME against the other stages of the DENV life cycle, we next analyzed the impact of the test drug on viral replication, translation, and egress. To this end, the cells were first infected with DENV before treatment with the test drug for 3 days and harvested for RNA and protein extraction. Quantitative PCR (qPCR) analysis revealed that the intracellular RNA content, which reflects DENV replication, was unaltered after the drug treatment (Fig. 6A). This observation was corroborated by results obtained from PCME treatment of Huh-7 replicon cells harboring intracellular replicating subgenomic DENV RNA (Huh7.D2-FLuc-SGR-Neo), which is a model that permits evaluation of the DENV intracellular replicative phase^18^. Specifically, PCME had no impact on the luciferase signal derived from the reporter-tagged DENV subgenomes, whereas ribavirin, a known inhibitor of DENV replication^18^, effectively suppressed the viral RNA production (Fig. 6B). Similarly, no significant impact was observed on the protein translation level wherein expression of DENV E, NS1, and NS5 proteins appeared unaltered with or without the drug treatment (Fig. 6C). To address whether PCME influenced virion release, we treated the DENV-infected Vero cells with the test drug for 3 days, before extracting extracellular proteins from the supernatant for Western blot analysis against the DENV E protein. As shown in Fig. 6D, DENV E protein was detected in the supernatant from DENV-infected cells (virus only). Treatment with PCME did not affect E protein content, with levels comparable to the control DMSO treatment (Fig. 6D). These results therefore indicated that PCME does not act on the DENV particle release, and together with the previous data suggested that PCME does not affect the other stages of the DENV life cycle, including replication, translation, and egress.

### Antiviral activity of PCME on DENV entry is not associated with influence on viral cell-to-cell spread

**Figure 7:**
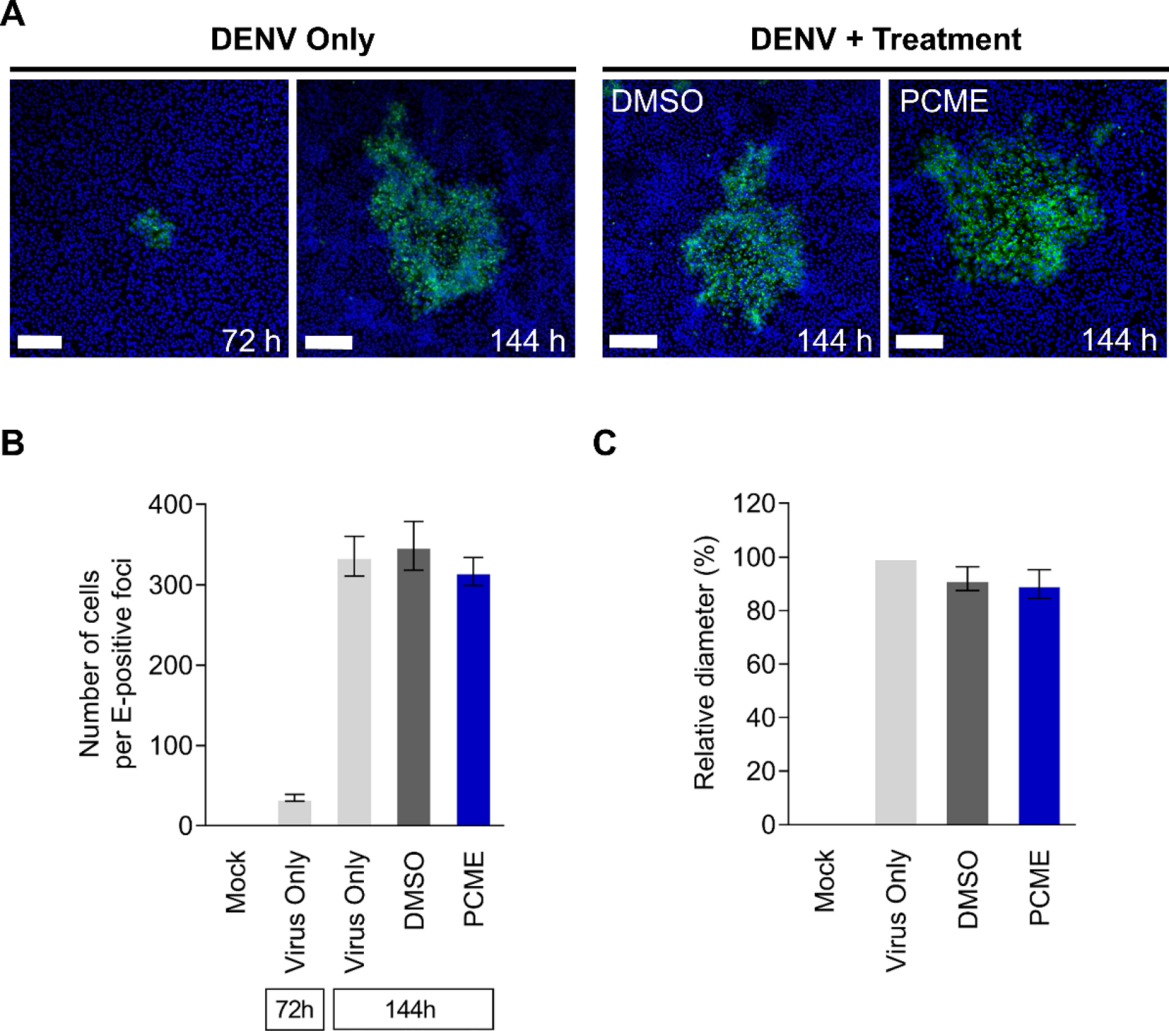
PCME does not inhibit DENV cell-to-cell spread. **(A)** Quantitative analysis of DENV cell-to-cell transmission in test drug-treated viral foci. Viral E protein (reflecting DENV-positivity) was detected by immunofluorescence microscopy at 72 h and 144 h postinfection. Scale bar = 200 μm. **(B)** Quantitation of the number of cells per DENV E-positive focus. Data were acquired from three randomly observed foci for each treatment group. **(C)** Determination of the relative diameter (%) of the DENV foci under test drug treatment compared to the virus only group at 144 h. PCME = 30 μg/ml; DMSO = 0.06%. Data shown are representative confocal micrographs or means ± SEM (*P < 0.05) from 3 independent experiments.

Based on our observation that PCME specifically targets DENV entry, we next investigated whether this natural phytobioactive extract could also block DENV postinfection cell-to-cell spread, an event that has been described previously^36^. Medium containing methylcellulose was used to overlay the DENV-infected cells, which prevented secondary infection from the released nascent cell-free virions but ensured viral transmission between intercellular junctions^19^. The DENV-positive foci would then be immunostained after PCME treatment. Following 3 days of incubation, established viral foci were visible, which grew in size at 6 days postinfection (Fig. 7A). Quantification from the number of cells per DENV-positive focus (stained with anti-DENV E antibody; Fig. 7B) and its measured diameter (Fig. 7C) revealed that PCME treatment resulted in similar DENV plaque sizes compared to the DMSO control, thus indicating that PCME’s anti-DENV activity has little or no influence on DENV cell–cell transmission. This observation explains why although PCME had an impact against DENV entry (Figs. 2C and 3), it had no effect upon postinfection treatment (Fig. 2D), since it was not able to inhibit the growth of established viral plaques from the methylcellulose-overlaid wells in our time-of-drug-addition assay, which only permitted cell-to-cell transmission but restricted secondary/de novo infections. These data therefore suggested that PCME’s antiviral activity against DENV entry does not implicate DENV cell-to-cell transmission.

### Impact of PCME against cell types relevant to DENV infection

**Figure 8:**
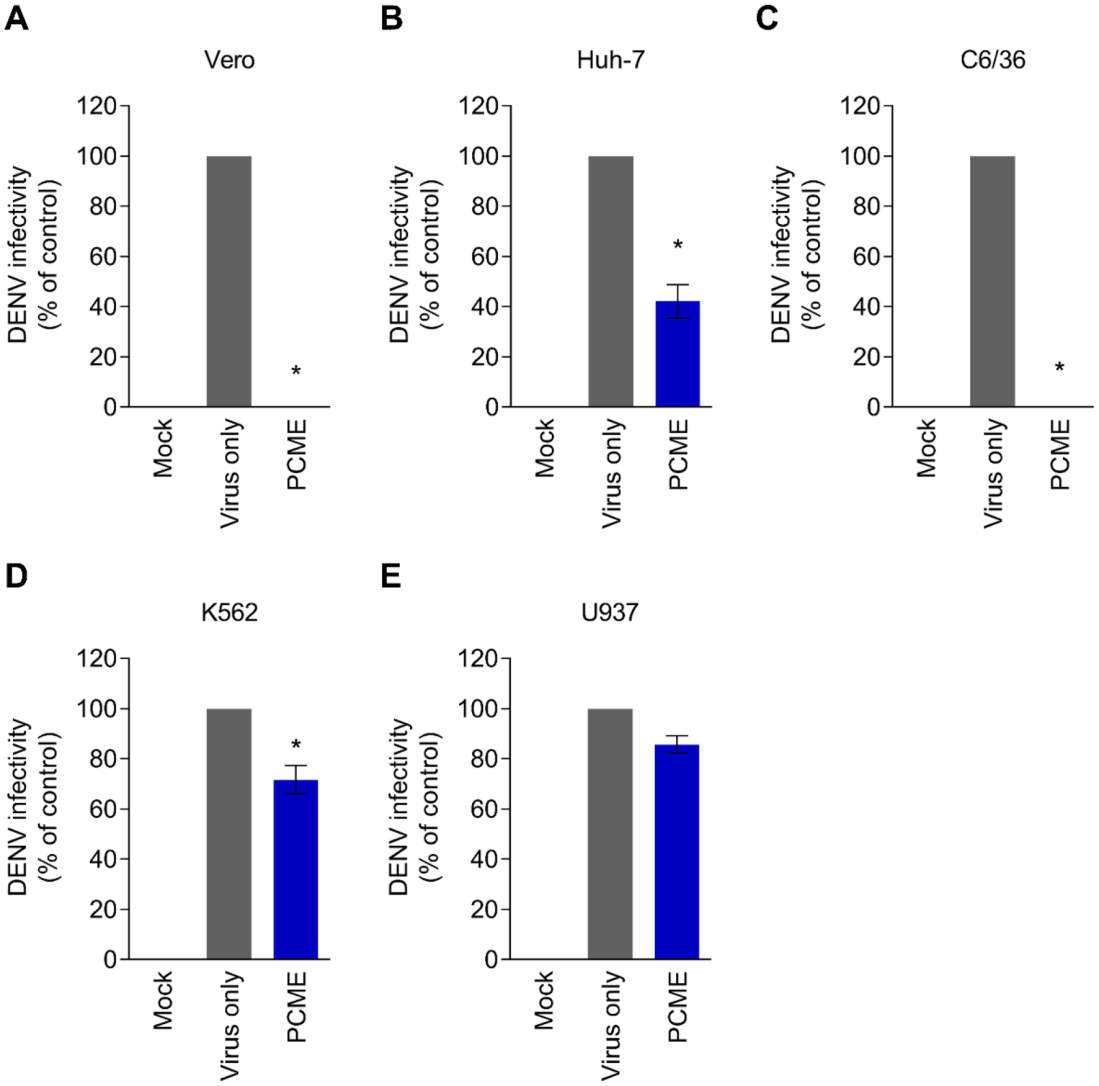
PCME impedes DENV infection of various cell types. Analysis of DENV infectivity in **(A)** Vero (DENV MOI = 0.1), **(B)** Huh-7 (DENV MOI = 1), **(C)** C3/C6 (DENV MOI = 0.1), **(D)** U937 (DENV MOI = 1), and **(E)** K562 (DENV MOI = 1) cells in the presence or absence of PCME treatment. For U937 and K562 cells, subneutralizing concentration (1: 100) of the 4G2 antibody was added at the time of infection to enhance the DENV infection via antibody enhancement. Following washes, the cells were incubated for 3 days prior to detection of DENV E protein by flow cytometry. Data shown are means ± SEM (*P < 0.05) from 3 independent experiments.

Next, we asked whether PCME’s ability to impede DENV entry is retained across cell types susceptible to DENV infection including Huh-7 (liver)^37^, C3/C6 (mosquito)^38^, U937 (myeloid)^27^, and K562 (erythroid)^28^ cells, by performing flow cytometry-based antiviral assay; Vero cells were included for comparison. Cells were treated concomitantly with DENV and the test drug, washed, and then further incubated until end-point before flow cytometry detection of DENV protein expression post viral entry. For U937 and K562, which are known to show low permissiveness to direct DENV infection^26–29^, antibody enhancement during the viral infection was used. Results indicated that PCME could efficiently impede DENV infection of the Huh-7 liver cells and the C6/36 mosquito cells similarly to its impact against the virus in Vero cells (Fig. 8A–C). However, the drug displayed minimal effect in restricting DENV infection of the monocytic U937 and erythroid K562 cells for which the DENV infection occurred via antibody enhancement (Fig. 8D,E). This disparity suggests that PCME’s anti-DENV effect could be dependent on the viral entry mechanism, and that it appears to be effective against direct DENV entry rather than antibody-mediated infection.

### Antiviral effect of PCME on the entry of other viruses in the Flaviviridae family

**Figure 9:**
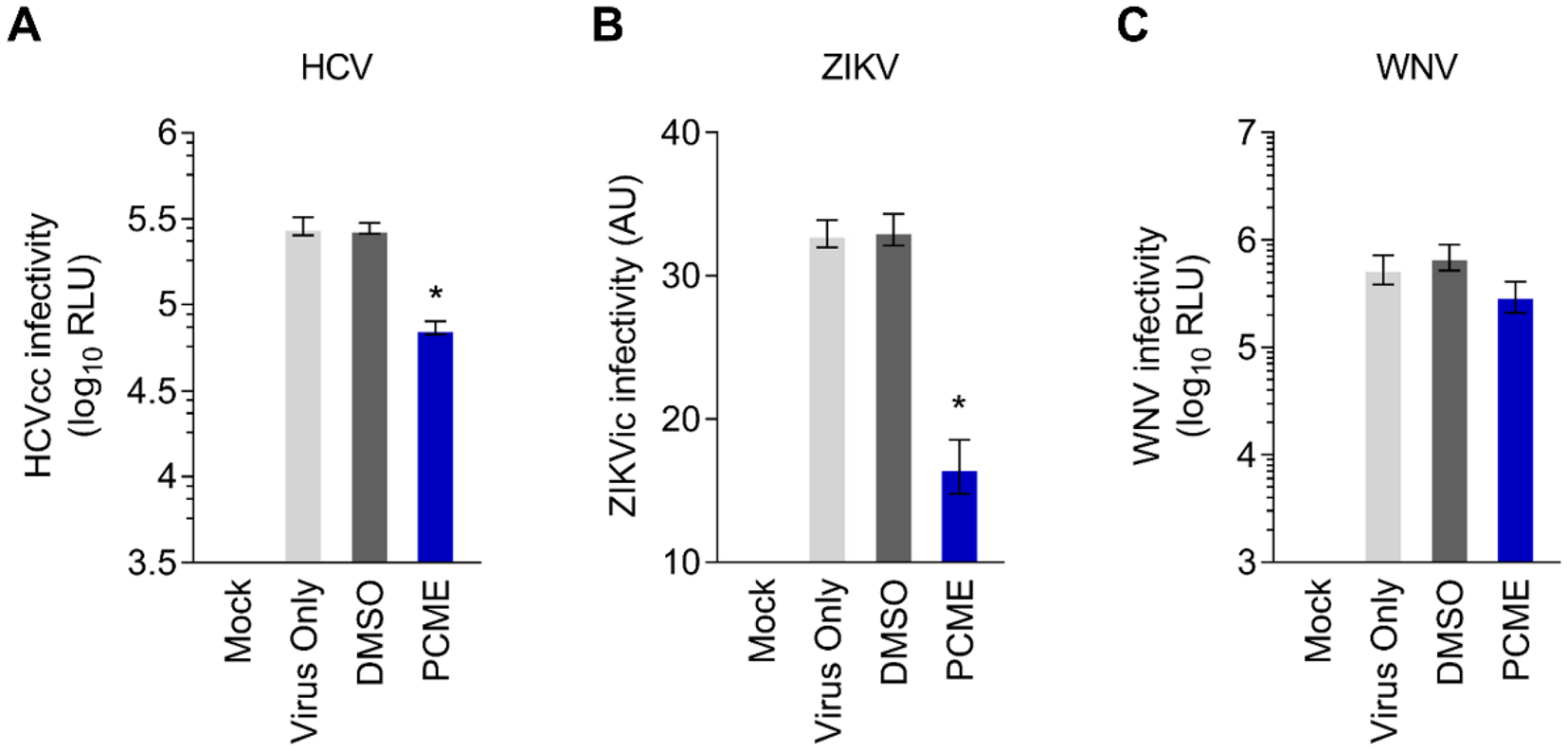
PCME inhibits the entry of HCV and ZIKV but not WNV. **(A)** Huh-7 cells seeded in 96-well plates were challenged with reporter-tagged HCVcc (MOI = 0.01) in the presence of 10 μg/ml PCME for 1.5 h. Cells were then washed with PBS and incubated in basal media for 72 h, before evaluating the *Gaussia* luciferase reporter activity. **(B)** Vero cells were seeded overnight in 6-well plates and infected with ZIKVic (MOI = 0.1) in the presence of 30 μg/ml of PCME for 2 h. The cells were then washed and incubated in growth media for 72 h, before being subjected to the flow cytometry-based infection analysis. **(C)** Vero cells seeded in 96-well plates were infected with WNV.RVPs (MOI = 0.1) for 2 h in the presence of 30 μg/ml PCME. The cells were subsequently washed and incubated in basal media for 72 h, before assessing the *Renilla* luciferase activity. Data shown are means ± SEM (*P < 0.05) from 3 independent experiments.

Given the robust antiviral activity of PCME on DENV entry, we finally sought to understand the effect of the phytobioactive material on some of the medically important viruses in the *Flaviviridae* family, including HCV, ZIKV, and WNV. To examine the antiviral effect of PCME against entry of these other viruses, cells were infected with the respective viral particles (reporter-tagged HCV, recombinant ZIKV, and reporter-tagged WNV) in the presence of PCME prior to further incubation and analysis. Our data indicated that in contrast to the DMSO control treatment, which yielded parallel results to the virus only group, the addition of PCME effectively hampered the entry of HCV (Fig. 9A) and even more significantly against ZIKV particles (Fig. 9B) as depicted by the drop in the viral infectivity the presence of the test drug. In contrast, PCME treatment had no significant impact on WNV infectivity as demonstrated by the comparable levels of viral infectivity with or without its presence (Fig. 9C). Together, these results suggest that, besides against DENV, PCME also possesses a broad-spectrum antiviral activity against selective viruses in the *Flaviviridae* family including HCV and ZIKV.

## Discussion

There are at present no clinically approved anti-DENV therapeutics, and a safe and effective immunization program is still under development. An anti-DENV treatment with prophylactic function could reduce the incidence of infection and help better manage future outbreaks in endemic areas. Our discovery that the methanolic extract of PC can robustly impede DENV entry by inactivating DENV free virus particles, blocking the viral attachment, and restricting viral entry/fusion highlights PC’s importance as a natural antiviral material against DENV infection. More importantly, our results imply that PCME could be further evaluated as an entry-specific antiviral candidate for developing effective prophylactic/therapeutic treatments against this arboviral infection.

Nature-derived materials represent an essential source of drug discovery. Recently, several natural ingredients displaying anti-DENV bioactivity have been identified, including the Brazilian coast marine seaweed *Palisada perforate*^39^, the natural alkaloid castanospermine^40^, cavinafungin derived from the fungus *Colispora cavincola*^41^, the natural β-carboline harmol^42^, the natural polyphenols delphinidin and epigallocatechin gallate ^43^, *Cissampelos pareira* Linn^44^, luteolin^45^, ecdysones from *Zoanthus* spp.^46^, and the latarcin peptide from *Lachesana tarabaeve* spider venom^47^. Our finding therefore adds PC as an important antiviral material source to the growing list of identified natural bioactive agents against DENV, which are useful for developing dengue treatment strategies.

Our data provided evidence that PCME possesses virucidal effect, being capable of rendering the cell-free DENV particles non-infectious (Fig. 3B). In addition, PCME could block DENV attachment (Fig. 3C) and the post-attachment viral entry/fusion event (Fig. 3D). We hypothesized that PCME might exert its antiviral activity by interacting with the surface structural components of DENV viral particles such as with the E envelope glycoprotein, which is responsible for cell adhesion and viral entry^34^. For instance, the drug could be interfering with DENV E-host receptor interactions and/or receptor engagement-induced conformational changes to the viral glycoprotein. In support of this hypothesis, we showed that PCME efficiently restricted the host cell entry of DENVpp (Fig. 5), which bear the DENV M and E on its surface, thus demonstrating the potential involvement of these surface architectural proteins in PCME’s anti-DENV activity. Alternatively, this natural phytobioactive material could also be virucidal (disrupting particle integrity) or could modify the host cell surface (such as by modulating DENV receptor expression) to preclude DENV entry. Further biophysical analysis of viral particles and host cell receptor expression examination are necessary to clarify the specific molecular mechanism(s) of action. Of note, while we identified PCME’s anti-DENV effect to be specifically targeting viral entry, it had no significant influence against viral cell-to-cell transmission (Fig. 7). Although DENV glycoprotein E has been suggested to be involved in the transmission of DENV progeny viral particles between cells in the absence of de novo entry^36^, it is possible that the viral machineries targeted by PCME in early viral entry and the later stage DENV intercellular spread are different or simply inaccessible to PCME in a spatio-temporal manner. Nonetheless, the observation that PCME’s anti-DENV entry activity does not implicate cell-to-cell transmission explains the loss of its effect against growth of DENV plaques in the postinfection treatment analysis (Fig. 2D), which was evaluated using methylcellulose overlay that excluded secondary/de novo infections, largely because the phytobioactive extract was unable to inhibit DENV intercellular spread from the already established infection. It could be interesting to use PCME to study the mechanistic differences in DENV entry with or without de novo infection.

Analysis of treatment effect on DENV entry in various cell types revealed that PCME could efficiently block direct DENV infection of relevant cells, including the Huh-7 liver cells and the C6/36 mosquito cells (Fig. 8B,C). In contrast, PCME exhibited minimal effect in restricting DENV infection in the monocytic (U937) and erythroid (K562) cells that required antibody enhancement (Fig. 8D,E)^27,28^. A plausible explanation for this discrepancy could be due to the difference in the mode of viral entry. While DENV enters Huh-7 and C6/36 cells mainly through receptor-mediated endocytosis, viral entry into the U937 and K562 cells in our antibody enhancement experimental model occurred via FcγR-mediated entry^34^. Thus, we hypothesized that PCME’s antiviral activity is only effective against direct DENV infection but not FcγR-mediated viral entry. Alternatively, it could also imply that antibody enhancement would weaken the antiviral efficacy of PCME in blocking DENV entry in these cells. Nonetheless, given that PCME blocked direct DENV infection in both human and mosquito cells (Fig. 8), it could potentially be further explored as an inhibitory agent for curbing mosquito acquisition of DENV or as therapeutics to diminish circulating DENV particles during dengue viremia, which is known to correlate with disease severity^48^. Additionally, restricting DENV infection will also considerably decrease risks of viral transmission from blood of infected individuals to mosquitoes, a phenomenon that could contribute to viral epidemic spread^49^.

Several major molecular constituents in PC have been identified, including resveratrol, quercetin, emodin, emodin-8-β-d-glucoside, and polydatin^8,50^. Although only resveratrol^51^ and quercetin^52^ were previously demonstrated to inhibit DENV infection by targeting post-infection viral replication, their influence on DENV entry is unclear. A preliminary attempt was made at deciphering the molecular bioactives responsible for PCME’s anti-DENV effect by treating the host cells with each of the major compounds from PCME specifically only during DENV inoculation as in Fig. 2C (‘Coaddition’ treatment model). While polydatin was observed to exert the most pronounced impact against DENV entry (Fig S1), its antiviral effect did not reach the same magnitude as the original extract itself (DENV infectivity reduction of ~ 47% versus ~ 99%, respectively). The other major compounds either showed little or no influence against DENV entry. This observation therefore suggests that additional small molecules or a combination of them might be responsible for the full magnitude of PCME’s anti-DENV effect, and further highlights the value of this natural phytobioactive extract as an excellent starting source for discovering novel anti-DENV entry inhibitors. An in-depth activity-based and fraction-guided analysis of PCME coupled by molecular signature identification should help with the above endeavors in identifying the molecular bioactives from PC.

Beside its robust inhibition of DENV infectivity, PCME also effectively blocked HCV and ZIKV entry, but not WNV entry (Fig. 9), implying that the natural agent possesses broad-spectrum antiviral activity against selective viruses in the *Flaviviridae* family. Notably, we observed that PCME was more potent in impeding ZIKV than HCV entry (Fig. 9A,B). The exact reason why PCME exerted a better antiviral effect against ZIKV than HCV or WNV is unclear but could be due to the greater genetic relatedness between DENV and ZIKV^53^. Indeed, studies have shown that the envelope glycoprotein E, which mediates viral entry and fusion, is highly conserved between the latter two viruses^54^, thereby potentially explaining why PCME demonstrated greater efficacy in impeding ZIKV infection compared to HCV and WNV. Since both ZIKV and DENV are transmitted by the same vector (*A. aegypti* mosquitos) and share similar geographic distribution, and given the current medical importance of ZIKV infection^55^, we suggest that PCME could also be of value as prophylaxis or starting point for developing preventive strategies against ZIKV infection.

In summary, our results demonstrated that PCME inhibits DENV infection by targeting the early viral entry events. The natural phytobioactive extract specifically neutralized the free viral particles and blocked both viral attachment and entry to the host cell cells, without exerting any significant influence on viral translation, replication, cell-to-cell spread, and release. Given its potential in restricting DENV infectivity, we suggest that PCME merits further exploration as a starting point antiviral source for developing anti-DENV prophylactics/therapeutics.

## Supplementary information

Supplementary information
