# Supplementary material for: Identification of the phytobioactive Polygonum cuspidatum as an antiviral source for restricting dengue virus entry
Source: Sci Rep. 2020 Oct 2;10:16378. doi: 10.1038/s41598-020-71849-3 (PMC7532532; doi:10.1038/s41598-020-71849-3)
Supplement: Supplementary file 1 — Supplementary information [file 41598_2020_71849_MOESM1_ESM.pdf]

Identification of the Phytobioactive *Polygonum cuspidatum* as an Antiviral Source for Restricting Dengue Virus Entry

Yu-Ting Kuo[a,1], Ching-Hsuan Liu[b,c,1], Jin-Wei Li[d], Chien-Ju Lin[e], Alagie Jassey[f], Huey-Nan Wu[g], Guey Chuen Perng[h,i], Ming-Hong Yen[d,e] and Liang-Tzung Lin[b,j,*]

[a] Department of Medical Imaging, Chi Mei Medical Center, Tainan, Taiwan

[b] Graduate Institute of Medical Sciences, College of Medicine, Taipei Medical University, Taipei, Taiwan

[c] Department of Microbiology & Immunology, Dalhousie University, Halifax, Nova Scotia, Canada

[d] Graduate Institute of Natural Products, College of Pharmacy, Kaohsiung Medical University, Kaohsiung, Taiwan

[e] School of Pharmacy, College of Pharmacy, Kaohsiung Medical University, Kaohsiung, Taiwan

[f] International Ph.D. Program in Medicine, College of Medicine, Taipei Medical University, Taipei, Taiwan

[g] Institute of Molecular Biology, Academia Sinica, Taipei, Taiwan

[h] Department of Microbiology and Immunology & Institute of Basic Medical Sciences, College of Medicine, National Cheng Kung University, Tainan, Taiwan

[i] Center of Infectious Diseases and Signaling Research, National Cheng Kung University, Tainan, Taiwan

[j] Department of Microbiology and Immunology, School of Medicine, College of Medicine, Taipei Medical University, Taipei, Taiwan

[1]These authors share first authorship.

***CORRESPONDENCE**

Liang-Tzung Lin, Department of Microbiology and Immunology, School of Medicine, College of Medicine, Taipei Medical University, No. 250, Wu-Hsing Street, Taipei 11031, Taiwan, Tel: +886-2-2736-1661 ext. 3911, Fax: +886-2-2736-1661 ext. 3921, E-mail: ltlin@tmu.edu.tw.

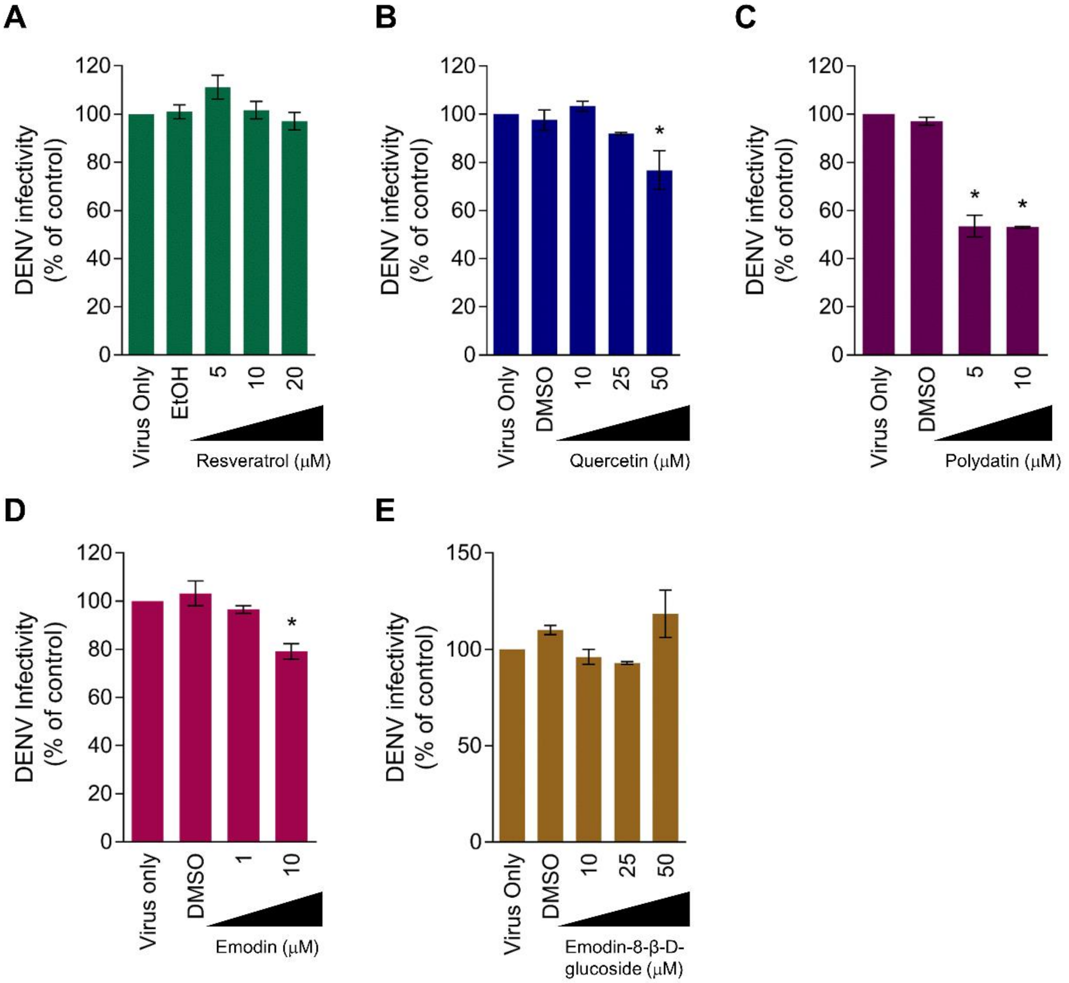

Fig S1. **Effect of major molecular components from PCME on DENV infectivity**. Vero cells (2 × 10^5 cells/well of 12-well plates) were infected with DENV-2 (100 PFU/well) in the presence of various concentrations of (A) Resveratrol, (B) Quercetin, (C) Emodin, (D) Polydatin, or (E) Emodin-8-β-D-glucoside for 1.5 h at 37 °C, washed with PBS, and incubated for 6 days. The cells were then immunostained with DENV E protein for viral plaque quantitation as described in the Materials and Methods. Data are plotted against the DMSO negative control treatment and expressed as percent (%) DENV infectivity. Data shown are means ± SEM (*$p < 0.05$) from 2 independent experiments.

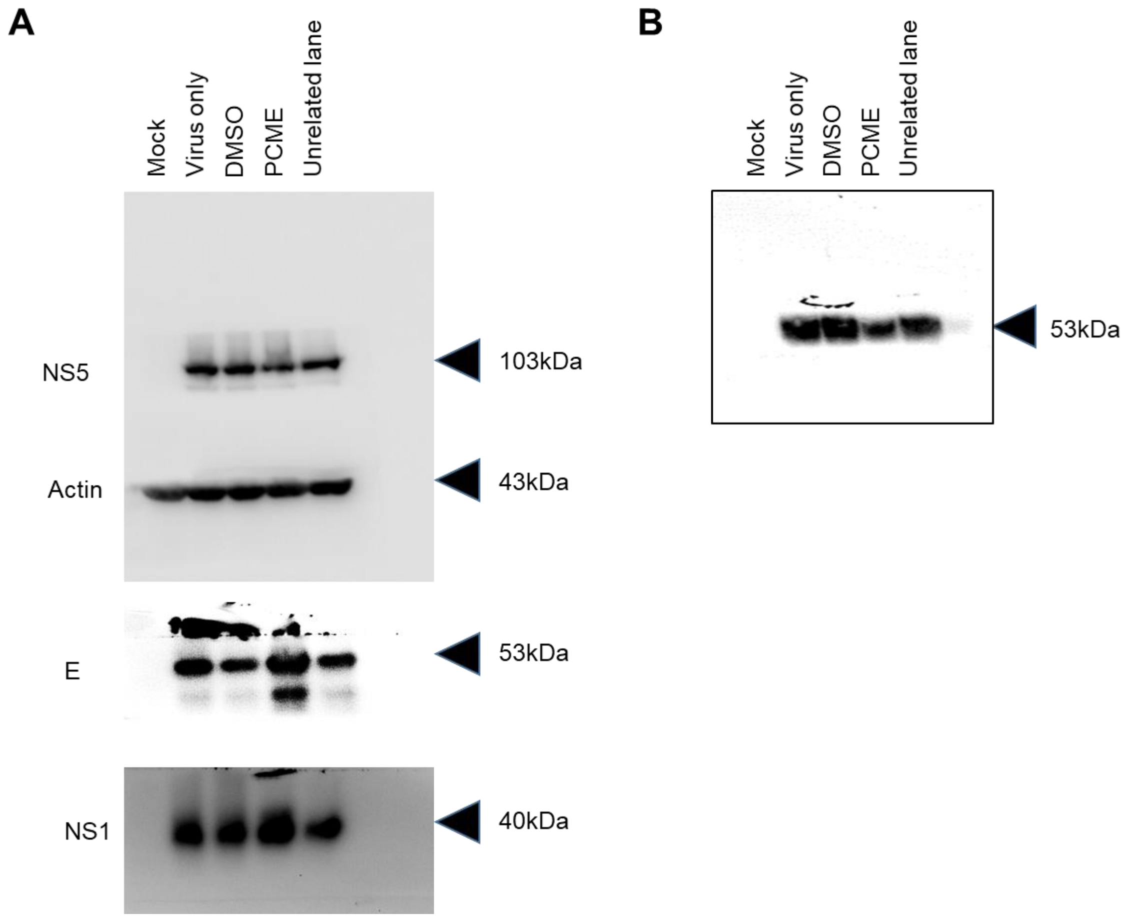

Fig S2. Full-length Western blots for the figures. (A) Figure 6C, **(B)** Figure 6D.
